# Spina bifida-predisposing heterozygous mutations in Planar Cell Polarity genes and Zic2 reduce bone mass in young mice

**DOI:** 10.1038/s41598-018-21718-x

**Published:** 2018-02-20

**Authors:** Isabel R. Orriss, Stuart Lanham, Dawn Savery, Nicholas D. E. Greene, Philip Stanier, Richard Oreffo, Andrew J. Copp, Gabriel L. Galea

**Affiliations:** 1 Department of Comparative Biomedical Sciences, Royal Veterinary College, Camden, London, NW1 0TU UK; 2 Bone and Joint Research Group, Centre for Human Development, Stem Cells and Regeneration, Human Development and Health, Institute of Developmental Sciences, Faculty of Medicine, University of Southampton, Southampton, SO16 6YD UK; 3 Developmental Biology of Birth Defects, UCL GOS Institute of Child Health, 30 Guilford Street, London, WC1N 1EH UK

## Abstract

Fractures are a common comorbidity in children with the neural tube defect (NTD) spina bifida. Mutations in the Wnt/planar cell polarity (PCP) pathway contribute to NTDs in humans and mice, but whether this pathway independently determines bone mass is poorly understood. Here, we first confirmed that core Wnt/PCP components are expressed in osteoblasts and osteoclasts *in vitro*. *In vivo*, we performed detailed µCT comparisons of bone structure in tibiae from young male mice heterozygous for NTD-associated mutations versus WT littermates. PCP signalling disruption caused by *Vangl2* (*Vangl2*^*Lp/*+^) or *Celsr1* (*Celsr1*^*Crsh/*+^) mutations significantly reduced trabecular bone mass and distal tibial cortical thickness. NTD-associated mutations in non-PCP transcription factors were also investigated. *Pax3* mutation (*Pax3*^*Sp2H/*+^) had minimal effects on bone mass. *Zic2* mutation (*Zic2*^*Ku/*+^) significantly altered the position of the tibia/fibula junction and diminished cortical bone in the proximal tibia. Beyond these genes, we bioinformatically documented the known extent of shared genetic networks between NTDs and bone properties. 46 genes involved in neural tube closure are annotated with bone-related ontologies. These findings document shared genetic networks between spina bifida risk and bone structure, including PCP components and Zic2. Genetic variants which predispose to spina bifida may therefore independently diminish bone mass.

## Introduction

Genetic analyses of rare human diseases associated with altered fracture risk have identified critical determinants of bone mass and architecture, some of which are now established clinical targets^1^. Fracture incidence in the general population follows a biphasic epidemiological pattern with a high incidence in childhood, a low incidence following skeletal maturity, increasing again in women following the menopause and in both men and women with age^2,3^. Progress has been made in delineating the genetic and pathophysiological basis of osteoporosis-related fragility fractures in the elderly^4,5^, whereas fractures during childhood have been less intensively studied. A number of genetic conditions presenting in childhood with primary skeletal disorders, such as osteogenesis imperfecta associated with mutations in the Wingless homology (Wnt) ligand Wnt1^6^, are now recognised, while multigenic disorders such as Down’s syndrome are also directly associated with skeletal effects^7^.

Paediatric fractures are important comorbidities in rare and multigenic conditions that do not primarily present with skeletal phenotypes, as is the case in paediatric spina bifida (myelomeningocele)^8–11^ patients. The best established risk factor for fractures in affected children is being non-ambulatory^9,10^, consistent with mechanical loading being the major functional determinant of bone mass and architecture. However, in a subset of patients primary skeletal disorders may also be involved. This is suggested by reports of fractures during childbirth and the neonatal period^12,13^ as well as low trauma fractures in ambulatory children^11^. Furthermore, differences in tibial mass and architecture in affected infants, independently of the degree of paralysis, have previously been described^14^. Specifically, tibiae from affected perinatal infants had smaller cortical area and thickness than age-matched infants who died for unrelated reasons, and their cross-sectional geometry clustered into groups with normal, increased or decreased eccentricity (a perfect circle has an eccentricity of 0)^14^.

Spina bifida and associated neural tube defects (NTDs) continue to affect around 1:1,000 pregnancies, with higher incidence in some geographical areas such as Northern China and in high risk groups such as those who have previously had an affected pregnancy^15,16^. Two molecular pathways clearly associated with spina bifida risk in humans are folate metabolism and the planar cell polarity (PCP) pathway, a non-canonical branch of Wnt signalling^16^. In mice, over two hundred genes have been identified which, when mutated, result in NTDs including spina bifida^17,18^. Some of these, such as components of the bone morphogenetic protein pathway^19^, are very well established to have critical skeletal roles independently of their role in neural tube closure. This raises the hypothesis that genetic variants which predispose to spina bifida may independently diminish bone mass. However the extent of the genetic commonalities between NTD risk and skeletal functional competence has not been systematically investigated.

Recently we reported that osteoblasts from NTD-predisposed *Loop tail* (*Lp*) mice which carry a heterozygous dominant negative mutation of Vangl2, a core PCP component, are less able to reorient their divisions following mechanical strain *in vitro*^20^. The PCP pathway is highly conserved from mammals to *Drosophila*, in which its core components follow highly polarized distributions in embryonic epithelia^21^. The pathway is activated by non-canonical Wnt ligands such as Wnt5a binding to the frizzled (Fzd) Wnt co-receptors. These cluster with the trans-membrane proteins Vangl1/2 and Celsr1-3, recruiting intracellular proteins including scribbled (Scrib) and dishevelled (Dvl). The outcomes of PCP signalling include regulation of gene expression through the JNK/c-Jun pathway as well as cytoskeletal reorganisation through Rho/ROCK signalling.

The roles of this pathway in mammals have primarily been studied in cancer and in development, primarily due to its role in convergent extension movements which narrow and elongate the early embryo^22^. Homozygous *Lp* mutation results in shorter but wider and thicker early limb buds and digit pre-chondrogenic condensates, as well as delayed ossification and absence of the middle phalanx in all digits^23^. This phenotype is exacerbated by heterozygous deletion of the PCP ligand Wnt5a, whereas homozygous Wnt5a deletion results in near-complete absence of digit mineralisation^23,24^. The roles of Wnt5a in osteoblasts and osteoclasts have been extensively studied. Its expression increases biphasically during osteoblastic differentiation^25^ and it promotes osteoclastic resorption in association with its alternate receptor, receptor tyrosine kinase-like orphan receptor 2^26^. However, whether its roles in postnatal bone include interactions with core PCP components are understudied.

We previously reported that Wnt5a mRNA increases rapidly in osteoblast-like cells subjected to strain *in vitro*^20^. *In vivo*, young female *Vanlg2*^*Lp/*+^ mice without spina bifida had lower distal femoral trabecular bone mass and a more circular mid-shaft cross-section than wild-type (WT) littermates^20^. This finding suggests that the PCP pathway influences post-natal bone mass and architecture and identifies a mouse genotype, known to be at increased risk of NTDs, which independently has low bone mass. This initial skeletal phenotyping was undertaken at a single pre-selected site of the femur, however, recent methodological developments now allow systematic analyses of cross-sectional bone mass and architecture across the bone’s length. For example, Site Specificity Analysis (SSA)^27^, has demonstrated that locally- and systemically-acting interventions can have site-specific effects on bone. SSA analysis has not yet been applied to document the effects of PCP pathway disruption on bone mass and architecture.

In this study we determined the expression of core PCP components during osteoblast and osteoclast differentiation *in vitro*. The relevance of this pathway to bone *in vivo* was investigated by applying SSA to study tibial structure in heterozygous *Lp* versus wild-type (WT) young male mice (assaying an additional bone and sex from that previously studied). To determine whether the roles of PCP signalling in bone extend beyond *Vangl2*, we similarly applied SSA to tibiae from *Crash (Crsh)* mice which carry a heterozygous mutation in another core PCP component, *Celsr1*, as well as in two non-PCP gene models, *Splotch* (*Sp*^*2H*^) and *Kumba* (*Ku*), which carry NTD-associated mutations in the transcription factors Pax3 and Zic2 respectively. Homozygous mutations of each of the genes tested result in NTDs with high or complete penetrance and render the mice non-viable, whereas a small proportion of heterozygous embryos develop spina bifida (*Vangl2*^*Lp/*+^ ~5%^28^, *Celsr1*^*Crsh/*+^ predisposed when modifier genes also mutated^29^, *Pax3*^*Sp/*+^ ~6%^30^, *Zic2*^*Ku/*+^ ~3%^31^). To determine whether commonalities extend beyond the genes directly investigated here, we subsequently document the known extent of shared genetic networks between NTD risk and bone-related functions.

## Results

### Core mammalian Wnt/PCP components are expressed in bone cells

**Figure 1:**
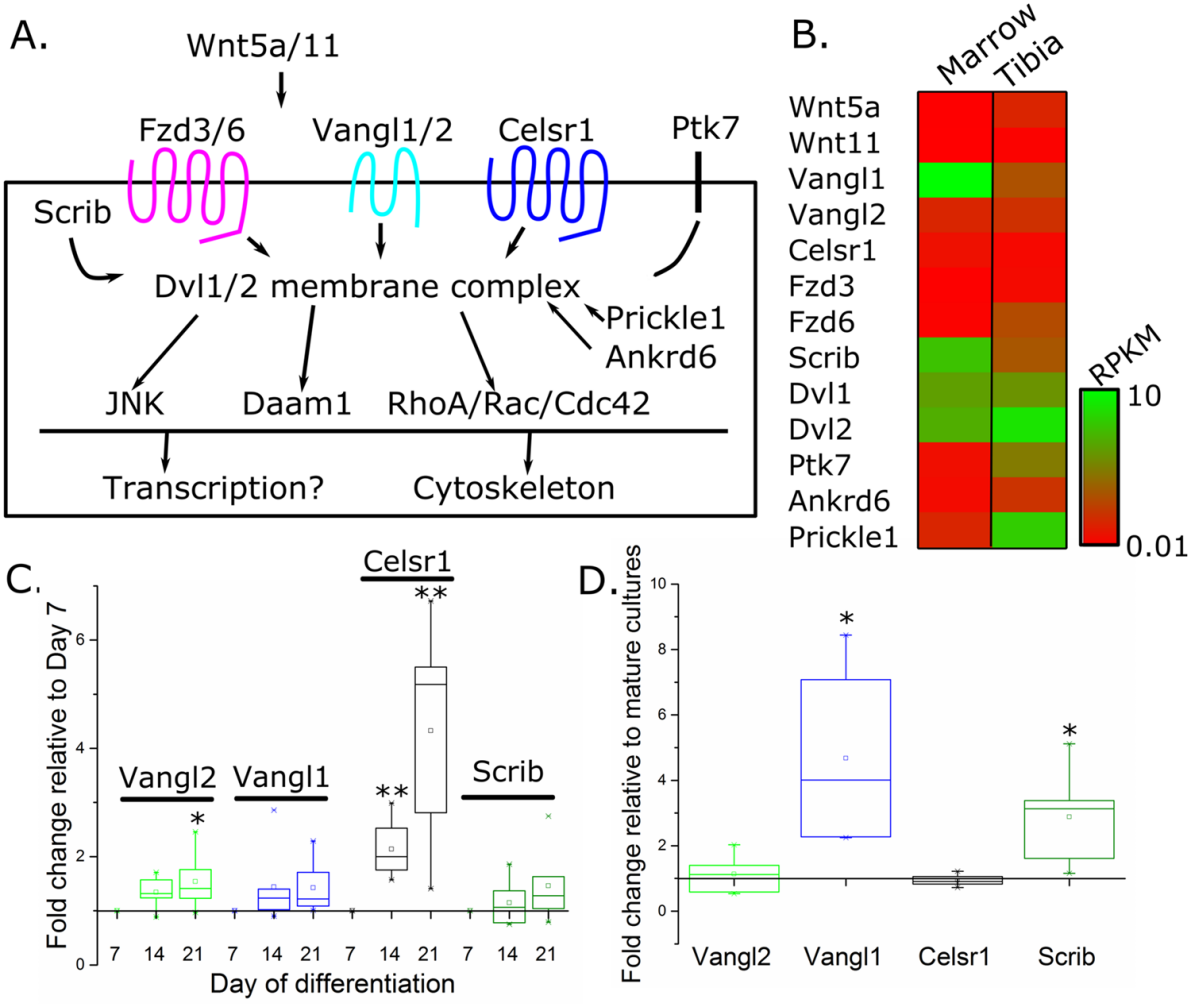
Core PCP pathway components are expressed in osteoblasts and osteoclasts. (**A**) Schematic illustration of components of the mammalian PCP pathway (based on Nikolopoulou *et al*.^21^). (**B**) Normalised detection levels of mammalian PCP components in bone marrow and tibial cortical bone from an RNA-Seq dataset previously reported by Ayturk *et al*.^32^. (**C**) qRT-PCR quantification of the core PCP components Vangl1, Vangl2, Celsr1 and Scrib in osteoblast cultures differentiated for 14 or 21 days, compared with osteoblasts cultured for 7 days (set at 1 for each set of cultures). (**D**) qRT-PCR quantification of Vangl1, Vangl2, Celsr1 and Scrib in mature resorbing osteoblasts versus mature osteoclasts (set at 1 for each culture). N = 4–6, *p < 0.05, **p < 0.01.

Core components of the mammalian Wnt/PCP pathway are expressed in osteoblasts and osteoclasts. Analysis of a previously-reported RNA-Seq dataset^32^ confirmed PCP pathway components are detectably expressed in bone and marrow of adult mice (Fig. 1A,B). In cultured osteoblasts, expression of *Vangl2* and *Celsr1* was increased in mature, mineralising osteoblasts (day 21) compared to differentiating cells (day 7). *Vangl1* and *Scrib* mRNA expression was not significantly altered during osteoblast differentiation (Fig. 1C). In contrast, *Vangl1* and *Scrib* mRNA expression was increased in mature, actively resorbing osteoclasts (day 9, two days following medium acidification) compared to mature, inactive osteoclasts (day 7). Vangl2 and Celsr1 mRNA expression was unaffected by osteoclast activation. This data demonstrates that core PCP components are expressed in osteoblasts and osteoclasts.

### Vangl2 mutation diminishes trabecular bone mass

**Figure 2:**
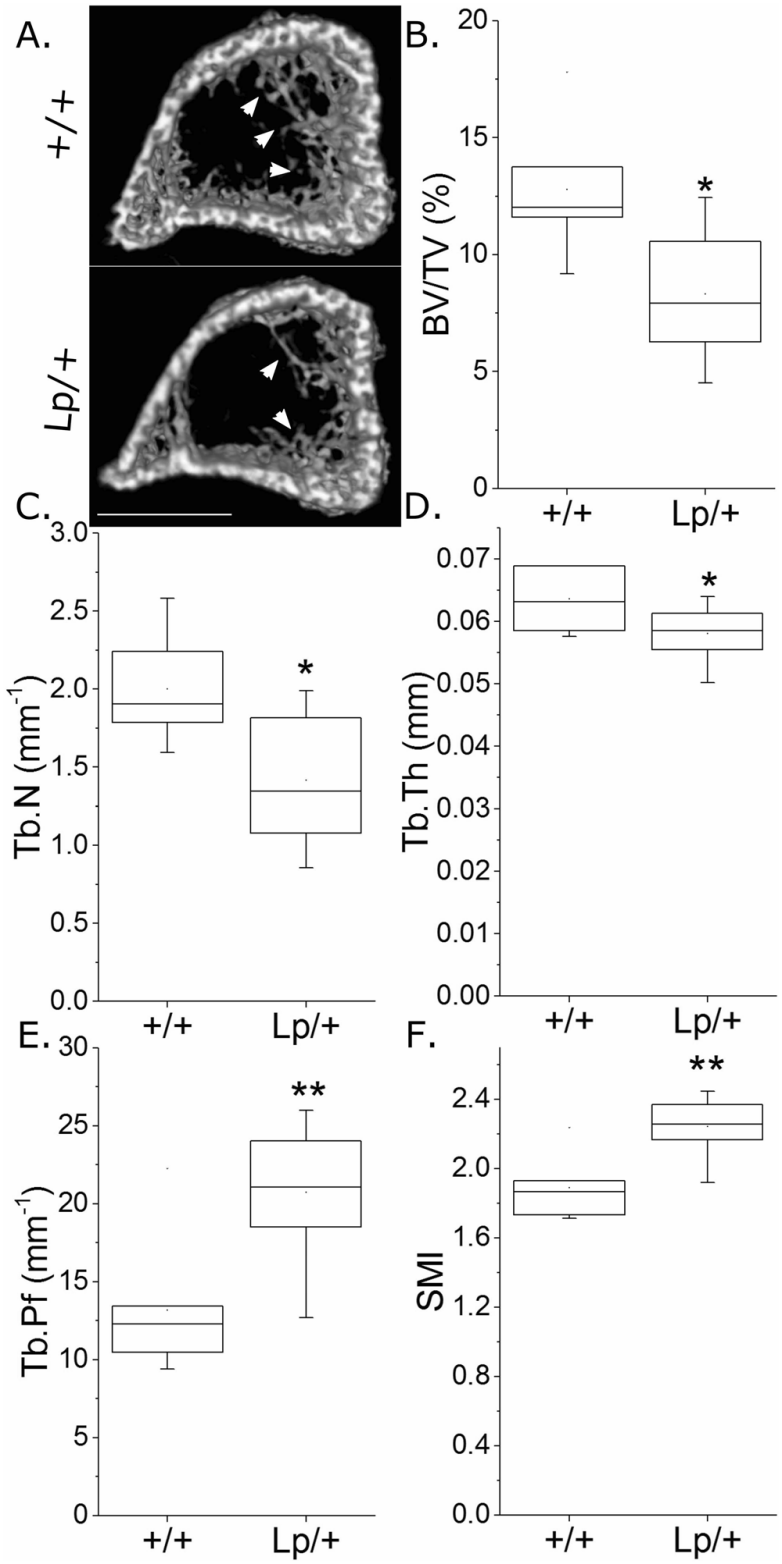
*Vangl2*^*Lp*/+^ mutation is associated with lower trabecular bone mass. µCT analysis of proximal tibial trabecular bone mass and architecture in 3-week-old male Vangl2^+/+^ and *Vangl2*^*Lp*/+^ littermates (n = 8 each). (**A**) Representative images of the proximal tibia showing less trabecular bone in the *Vangl2*^*Lp*/+^ tibia (arrows). Scale bar = 1 mm. B) BV/TV, (**C**) Tb.N, (**D**) Tb.Th, (**E**) Tb.Pf and (**F**) SMI were compared. *p < 0.05, **p < 0.01 by independent samples t-test.

Male mice carrying a heterozygous *Vangl2* mutation, *Vangl2*^*Lp/*+^, displayed diminished trabecular bone mass. Young male *Vangl2*^*Lp/*+^ mice had lower bone volume per tissue volume (BV/TV, −35.0% on average), trabecular number (Tb.N, −29.2%) and trabecular thickness (Tb.Th, −8.7%), and higher trabecular pattern factor (Tb.Pf, ^+^5.7%) and structure model index (SMI, ^+^18.7%) than *Vangl2*^+*/*+^ littermates (Fig. 2A–F). Trabecular separation (Tb.Sp) was unchanged (Supplementary Table S1).

**Figure 3:**
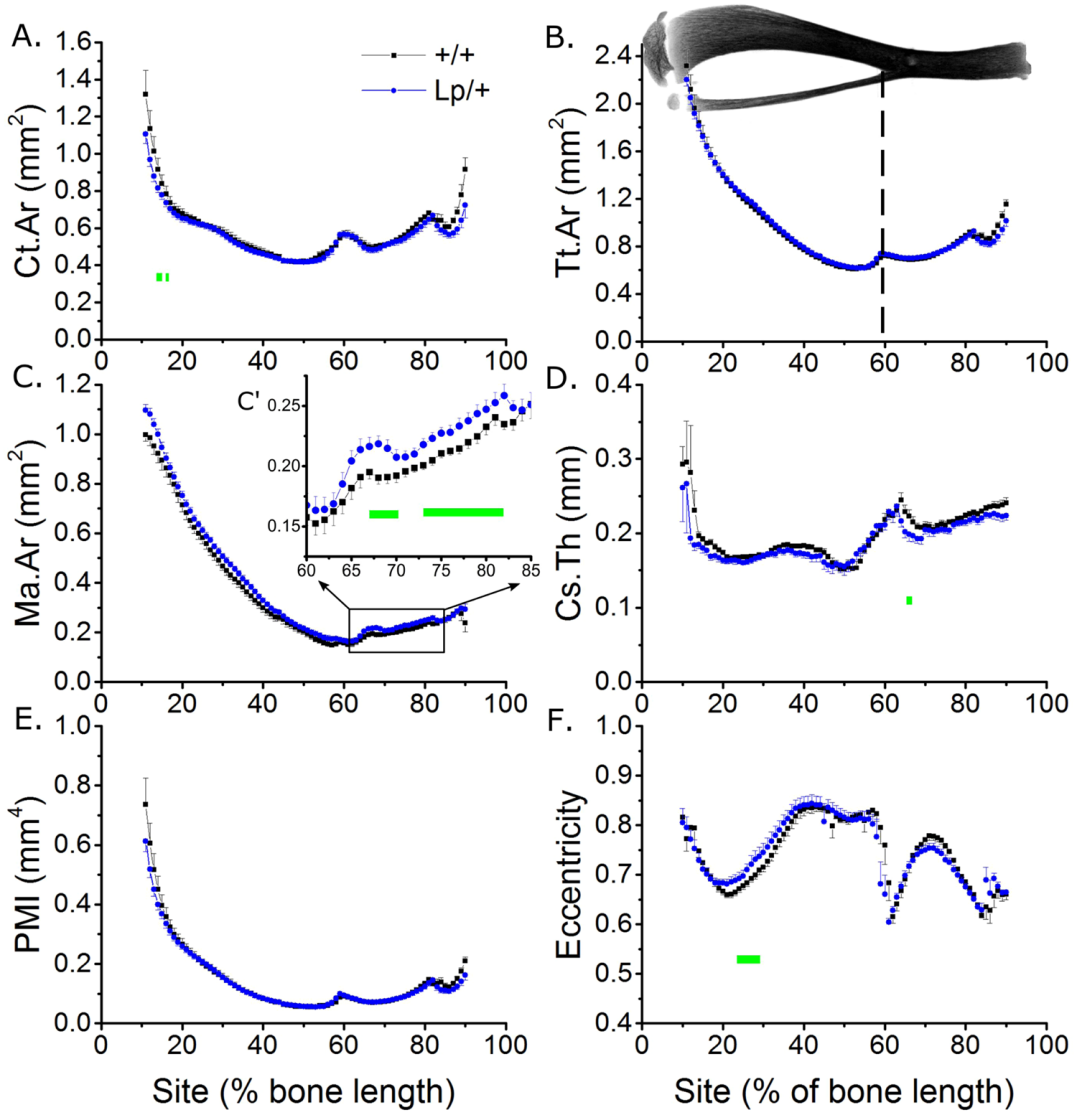
*Vangl2*^*Lp*/+^ mutation is associated with regionally smaller cortical bone mass in the tibia of young male mice. SSA µCT analysis of cortical bone structure at each 1% site along the bone’s length in 3-week-old male Vangl2^+/+^ and *Vangl2*^*Lp*/+^ littermates (n = 8 each). The tibia image indicates the orientation of SSA (proximal at 0% to distal at 100%). The tibia/fibula junction is indicated by the dashed vertical line (the fibula is automatically excluded from SSA). (**A**) Ct.Ar, (**B**) Tt.Ar, (**C**) Ma.Ar, (**D**) Cs.Th, (**E**) PMI and (**F**) eccentricity were quantified. (C’) Magnified view of marrow area quantification in the distal tibia. Green lines indicate regions of significant differences between genotypes by mixed model analysis with Bonferroni post-hoc.

Detailed analysis of tibial cortical bone using SSA identified minimal effects of *Vangl2*^*Lp/*+^ mutation on cortical bone mass (Fig. 3). Cortical area (Ct.Ar) was 3.3% lower on average across all sites in tibiae from *Vangl2*^*Lp/*+^ than *Vangl2*^+*/*+^ mice (Fig. 3A) whereas total tissue area (Tt.Ar) was not significantly different (Fig. 3B). Medullary area (Ma.Ar) was bigger (+7.8% across all sites, Fig. 3C) whereas cross-sectional thickness (Cs.Th) was smaller (−3.7% across all sites, Fig. 3D) on average in tibiae from *Vangl2*^*Lp/*+^ mice. These differences were not associated with a significant change in polar moment of inertia (PMI, a computed parameter associated with bone strength, Fig. 3E). Although none of the differences overserved were site-specific (genotype * site interaction p > 0.10 in each case), the differences in Ct.Ar reached significance in the proximal tibiae (Fig. 3A) whereas differences in Ma.Ar and Cs.Th were significant at sites distal to the tibia/fibula junction (Fig. 3C,D).

Eccentricity, which is a measure of cross-sectional circularity rather than mass, was significantly higher in the proximal tibia of *Vangl2*^*Lp/*+^ than *Vangl2*^+*/*+^ mice (Fig. 3F).

### Celsr1 mutation diminishes trabecular bone mass and mid-shaft cortical thickness

**Figure 4:**
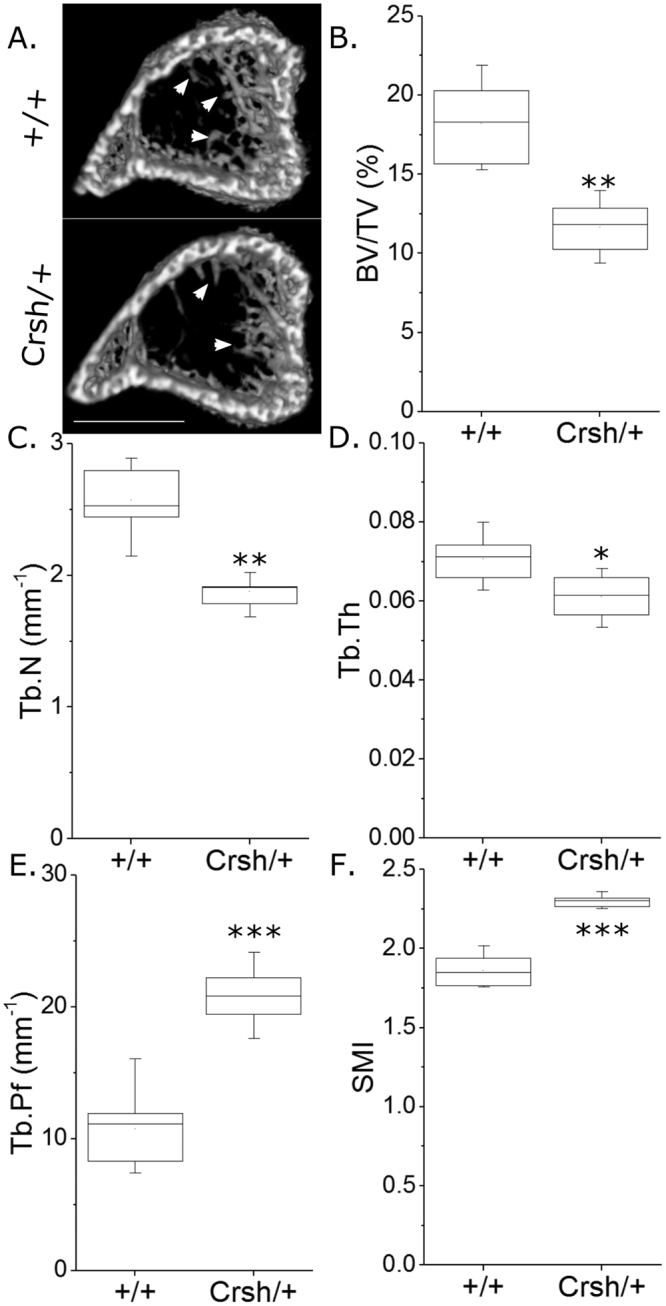
*Celsr1*^*Crsh*/+^ mutation is associated with lower trabecular bone mass. µCT analysis of proximal tibial trabecular bone mass and architecture in 3-week-old male Celsr1^+/+^ and *Celsr1*^*Crsh*/+^ littermates (n = 8 each). (**A**) Representative images of the proximal tibia showing less trabecular bone in the *Celsr1*^*Crsh*/+^ tibia (arrows). Scale bar = 1 mm. (**B**) BV/TV, (**C**) Tb.N, (**D**) Tb.Th, (**E**) Tb.Pf and (**F**) SMI were compared. *p < 0.05, **p < 0.01 by independent samples t-test.

Mice carrying the heterozygous Celsr1 mutation, *Celsr1*^*Crsh/*+^, displayed diminished trabecular bone mass, similarly to that seen in heterozygous *Loop tail* mice, demonstrating other components of the PCP signalling pathway also contribute to this bone compartment. Specifically, young *Celsr1*^*Crsh*/+^ male mice had lower BV/TV (−41.0%), Tb.N (−23.5%) and Tb.Th (−15.5%), and higher Tb.Pf (+44.4%) and SMI (+16.5%) than *Celsr1*^+*/*+^ mice (Fig. 4A–F and Supplementary Table S2).

**Figure 5:**
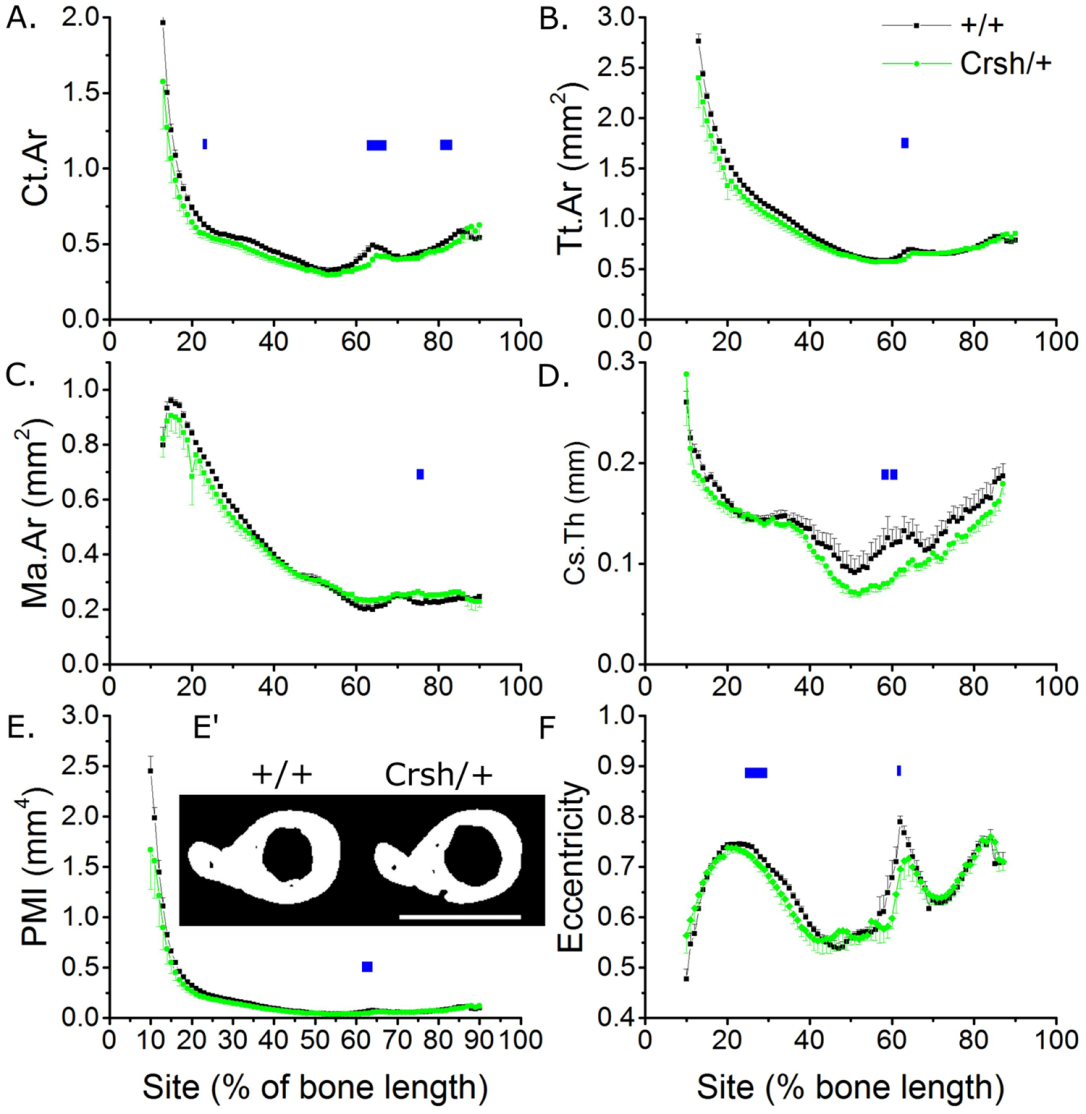
*Celsr1*^*Crsh*/+^ mutation is associated with regionally smaller cortical bone mass in the tibia of young male mice. SSA µCT analysis of cortical bone structure at each 1% site along the bone’s length in 3-week-old male Celsr1^+/+^ and *Celsr1*^*Crsh*/+^ littermates (n = 8 each). (**A**) Ct.Ar, (**B**) Tt.Ar, (**C**) Ma.Ar, (**D**) Cs.Th, (**E**) PMI and (**F**) eccentricity were quantified. (E’) µCT cross-sections through the tibia immediately distal to the tibula/fibula junction (62% site). Scale bar = 1 mm. Blue lines indicate regions of significant differences between genotypes by mixed model analysis with Bonferroni post-hoc.

In the tibial cortical compartment, the *Crsh* mutation in *Celsr1* was associated with smaller Ct.Ar and Tt.Ar compared with *Celsr1*^+*/*+^ littermates (−11.0% and −5.7% on average across all sites respectively, Fig. 5A,B). Ma.Ar was regionally higher (Fig. 5C) in the same region of the tibia affected by mutation of *Vangl2*. The *Crsh* mutation had a more pronounced effect on Cs.Th (−14.7% across all sites), reducing it primarily in the distal tibia below the tibia-fibula junction, and consequently reducing PMI (−14.0% across all sites) significantly in the same region (Fig. 5D,E,E’). None of the differences observed were site-specific (genotype * site interaction p > 0.10 in each case).

Contrary to the effect of the *Lp* mutation, *Crsh* reduced eccentricity (Fig. 5F).

### Mutation of the non-PCP gene, Pax3 minimally affects bone mass

*Pax3* mRNA expression was below the limit of reading in osteoblast and osteoclast cultures (C_T_ > 35 cycles whereas C_T_ values in E9.5 mouse embryo RNA used as positive controls were 26.3 ± 0.7 cycles, mean ± SEM, n = 3). Consistent with this, heterozygous mutation of Pax3 (*Pax3*^*Sp2H/*+^) in the *Splotch* mouse, did not significantly alter trabecular bone structure and minimally affected cortical bone in the tibia of young male mice. None of the standard measures of trabecular bone mass and architecture were significantly different between *Pax3*^*Sp2H/*+^ and *Pax3*^+*/*+^ littermates (Supplementary Table S3).

Ct.Ar, Tt.Ar and PMI were unchanged between *Pax3*^*Sp2H/*+^ and *Pax3*^+*/*+^ littermates, whereas Ma.Ar was significantly larger and Cs.Th was smaller (+4.8% and −7.1% on average across all sites respectively), primarily in a localised region of the distal tibia Supplementary Figure S1). None of the differences observed were site-specific (genotype * site interaction p > 0.10 in each case).

Unlike the PCP mutants analysed above, eccentricity was not different between *Pax3* genotypes (Supplementary Figure S1).

### Mutation of the non-PCP gene Zic2 diminishes cortical bone structure

*Zic2* mRNA expression increased in mineralising (day 21) relative to differentiating (day 7) osteoblasts and tended to increase in resorbing (day 9) versus mature (day 7) osteoclasts (Supplementary Figure S2).

**Figure 6:**
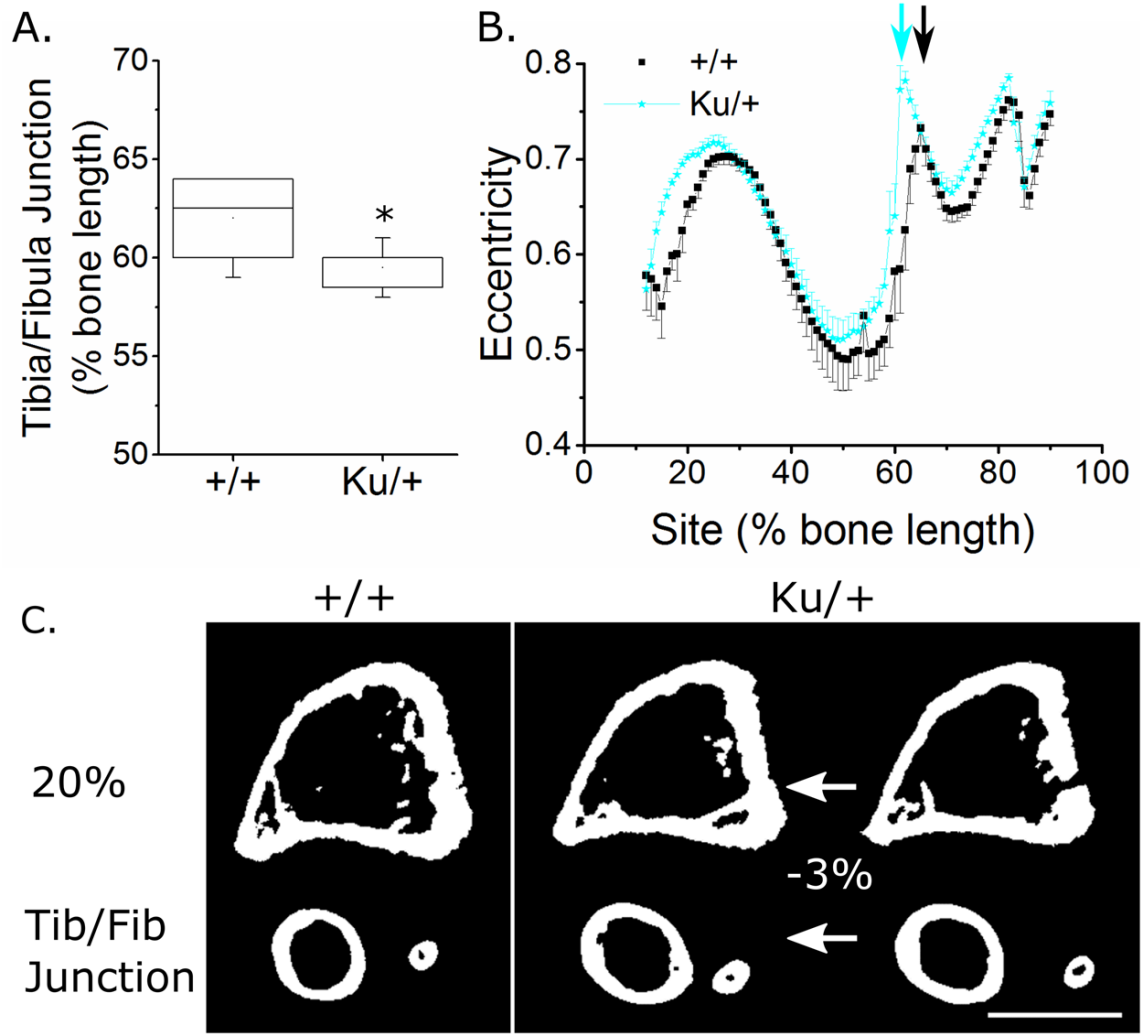
Heterozygous *Zic2*^*Ku*/+^ mutation is associated with a more proximally located tibia/fibula junction. (**A**) Quantification of the tibia/fibula junction as a percentage of bone length in Zic2^+/+^ and *Zic2*^*Ku*/+^ littermates (n = 8 each). (**B**) SSA analysis of eccentricity indicating the apparent shift in eccentricity associated with the more proximally-located tibia fibula junction in *Zic2*^*Ku*/+^ (cyan arrow) than Zic2^+/+^ (black arrow) littermates. (**C**) µCT cross-sections showing tibial structure at the 20% site and at the tibia/fibula junction in Zic2^+/+^ mice. The *Zic2*^*Ku*/+^ cross-sections are shown at the same percentage sites as the Zic2^+/+^ on the right and normalised to the position of the tibia-fibula junction (shifted by 3% of the bone’s length) on the left. Comparisons between Zic2^+/+^ and *Zic2*^*Ku*/+^ mice shown here were made following normalisation to the position of the tibia/fibula junction, but general reduction in cortical bone mass holds true if no correction is applied.

Heterozygous mutation of Zic2 (*Zic2*^*Ku/*+^) in the *Kumba* (*Ku*) mouse diminished cortical bone mass without altering trabecular bone in the tibia of young male mice. None of the standard measures of trabecular bone mass and architecture assessed were significantly different between *Zic2*^*Ku/*+^ and Zic2^+/+^ littermates (Supplementary Table S4). In cortical bone, SSA revealed that the tibia/fibula junction was significantly more proximal, as a percentage of the bone’s length, in *Zic2*^*Ku/*+^ than littermate *Zic2*^+*/*+^ (2.5% more proximal on average, Fig. 6A). Tibial length was unchanged (Supplementary Table S4). The shift in tibia/fibula junction position is clearly visible in SSA plots of tibial eccentricity, which show a sharp change at the point the tibia and fibula become separated (Fig. 6B). To account for this, the analysis of each tibia was adjusted such that the tibia/fibula junction was defined as being at 61% of the bones length (average junction position across the two genotypes). Applying this normalisation reduced the observed differences in cortical bone mass (e.g. at the 20% site of the proximal tibia, Ct.Ar was on average 34% lower in *Zic2*^*Ku/*+^ than littermate *Zic2*^+*/*+^ prior to normalisation and 27% lower following normalisation).

**Figure 7:**
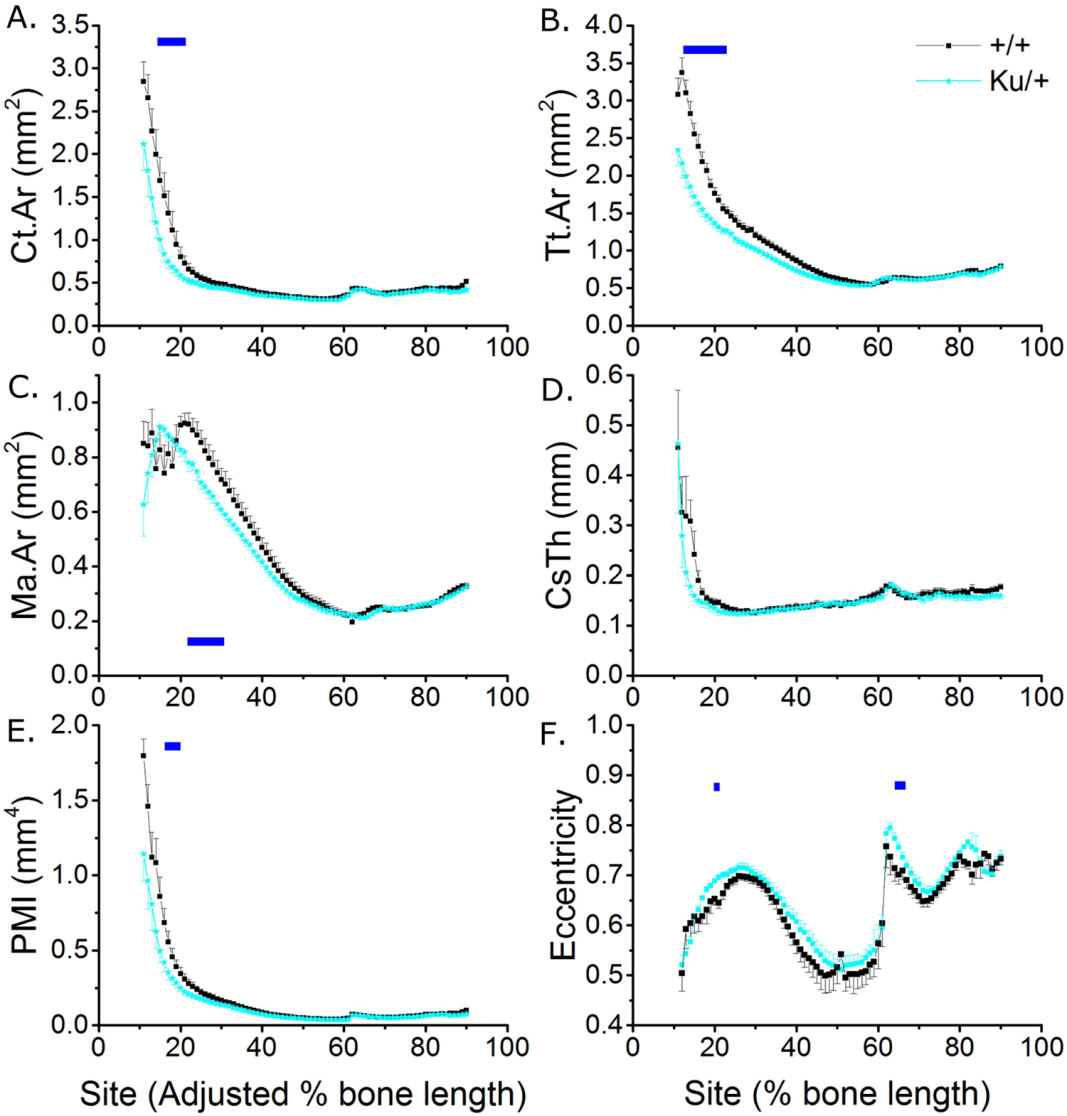
*Zic2*^*Ku*/+^ mutation is associated with significantly smaller cortical bone mass in the proximal tibia of young male mice. SSA µCT analysis of cortical bone structure at each 1% site along the bone’s length in 3-week-old male Zic2^+/+^ and *Zic2*^*Ku*/+^ littermates (n = 8 each). (**A**) Ct.Ar, (**B**) Tt.Ar, (**C**) Ma.Ar, (**D**) Cs.Th, (**E**) PMI and (**F**) eccentricity were quantified. Blue lines indicate regions of significant differences between genotypes by mixed model analysis with Bonferroni post-hoc.

Following normalisation young male *Zic2*^*Ku/*+^ mice had lower Ct.Ar (−13.4% across all sites), Tt.Ar (−12.0% across all sites) and Ma.Ar (−6.5% across all sites) than *Zic2*^+*/*+^ littermates, with differences reaching significance at various sites in the proximal tibia (Fig. 7A–C). The reduction in Tt.Ar was site specific (site * genotype interaction p = 0.045). Cs.Th was unchanged, but PMI was smaller (−17.6% across all sites) in tibiae of *Zic2*^*Ku/*+^ mice (Fig. 7D,E).

Although normalising the position of the tibia/fibula junction largely restored the otherwise marked apparent effect of the *Zic2* mutation, eccentricity was nonetheless greater compared with *Zic2*^+*/*+^ littermates in the proximal tibia and immediately distal to the tibia/fibula junction (Fig. 7F).

### Interlinked networks of genes involved in neural tube closure have skeletal functions

**Figure 8:**
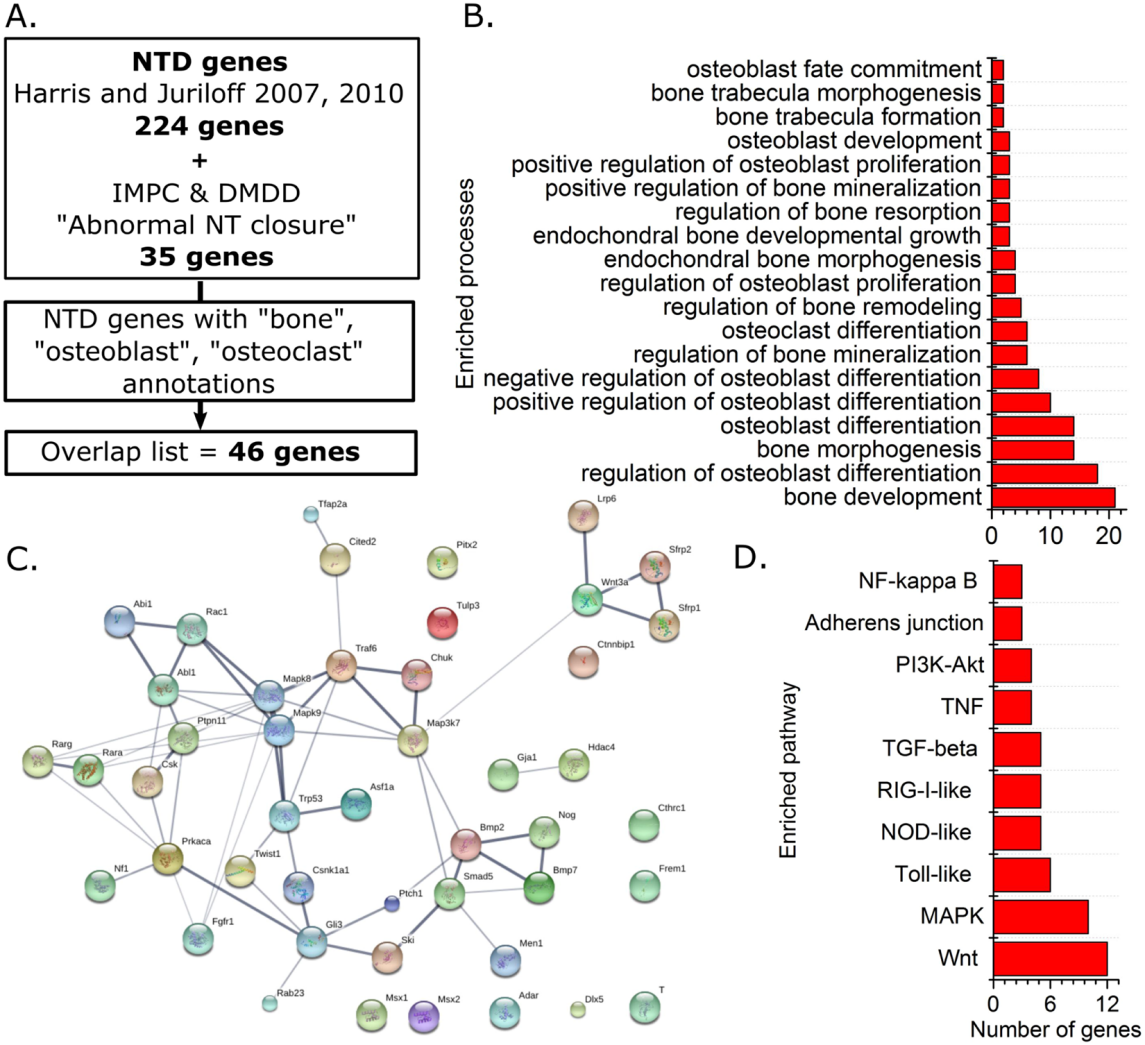
Genetic commonalities between neural tube defects and bone-associated genes are enriched for pathways with critical skeletal functions. (**A**) Schematic of the processes by which the extent of known genetic commonality was determined. (**B**) “Bone”, “osteoblast” and “osteoclast” associated gene ontology terms enriched in the NTD genes list. (**C**) LinkDB map of interacting genes in the Overlap list. (**D**) Signalling pathways enriched in the Overlap list.

NTD determinants and bone-related functions share genetic networks which extend beyond PCP signalling and Zic2. 259 genes were identified to be involved in mouse neural tube closure based on literature reviews published by Harris and Juriloff^17,18^ and genotypes annotated as having “abnormal neural tube closure” by the International Mouse Phenotyping Consortium or Deciphering Mechanisms of Developmental Disorders consortia (as of May 2017, Fig. 8A). This NTD genes list is significantly enriched for biological processes containing “bone”, “osteoblast” or “osteoclast” terms (p < 0.05 in each case, Fig. 8B). These terms are associated with 46 genes (Supplementary Table S5) that indicate genetic commonalities between NTD phenotypes and skeletal functions. These 46 genes form an interlinked network (Fig. 8C) of genes with well-known functions in bone including secreted ligands or their receptors (e.g. Fgfr1, Bmp2, Wnt3a), intracellular signalling molecules (e.g. Rac1, Mapk8) and transcriptional regulators (e.g. Rara/g, Msx1, Dlx5). Indeed, various signalling pathways with key functions in bone are significantly over-represented in this list (Fig. 8D). The most over-represented pathway is Wnt signalling (12 genes, adjusted p < 1 × 10^−13^), including known components of the canonical Wnt pathway such as Wnt3a and Lrp6. Core components of the PCP pathway and Zic2 did not feature, reflecting the current lack of knowledge of these genes’ functions in bone.

## Discussion

Here we tested the hypothesis that mouse genotypes predisposed to spina bifida independently have diminished bone mass. Hypothesis based selection of genes with well-established roles in neural tube closure confirmed that the PCP pathway contributes to trabecular bone mass, as well as implicating *Zic2* in the determination of cortical bone mass. Unbiased bioinformatics analysis of genes known to be involved in neural tube closure identified 46 genes with annotated roles in bone, primarily involving bone development and osteoblast functions. This demonstrates genetic commonalities, at least in mice, between NTDs and bone properties. Implied functional commonalities between NTDs and bone competence may compound with other fracture risk factors such as disuse and vitamin D deficiency in spina bifida patients^9–11^.

The importance of PCP signalling to skeletal development is clearly shown by limb dysmorphogenesis in *Vangl2*^*Lp/Lp*^ embryos, *Vangl2*/*Wnt5a* double mutant embryos, and *Prickle1* mutants^23,24,33^. However, the post-natal roles of PCP and specifically Vangl2 in bone are poorly understood. In order to confirm the potential relevance of PCP signalling to bone biology we first confirmed expression of several known core pathway components. While *Vangl1* and *Scrib* were expressed at stable levels during osteoblastic differentiation *in vitro*, expression of both *Vangl2* and *Celsr1* increased significantly with differentiation and the onset of mineralisation. This is consistent with a previous report that deletion of the PCP downstream component Jnk1 diminishes late osteoblastic differentiation *in vitro* and decreases bone formation *in vivo*^34^. In contrast, *Vangl1* and *Scrib* expression increased in resorbing osteoclastic cells *in vitro*. What functions of these cells are promoted or inhibited by the PCP pathway is not known, but roles of this pathway in other cell types include coordination of cell migration^35,36^, extracellular matrix remodelling^37^, and organisation of the actin cytoskeleton^38,39^. This is consistent with our previous report that prevention of strain-related actin regulation by the PCP down-stream component ROCK, or mutation of *Vangl2* in cortical osteoblasts from *Lp* mice, in each case prevented reorientation of cell division^20^. Of note, Vangl2 protein follows a planar-polarised distribution along the proximal to distal axis of developing chondrocytes and WT Vangl2 protein retains this distribution in heterozygous *Vangl2*^*Lp/*+^ fetuses^40^, despite the *Lp* allele having dominant negative effects in other contexts^41^.

Analyses of *Lp* mice have delineated roles for Vangl2 in various organs including the lungs^42^ and kidneys^43^, in which it is involved in branching morphogenesis. It is therefore intriguing that *Vangl2* mutation primarily diminishes bone mass in the branched, trabecular compartment, particularly as both of the PCP mutant genotypes investigated here primarily had a reduced number of trabeculae. This finding is consistent between the femur of female mice previously reported^20^ and the tibia of male mice studied here. *Vangl2* mutation had minimal effects in the cortical compartment, which was extensively mapped using SSA in the present study thereby providing a more global representation of their skeletal phenotype than can be achieved by analysing individual sites. However, whereas *Vangl2* mutation decreased eccentricity in the femoral midshaft^20^, it resulted in greater eccentricity in the proximal tibia. This discrepancy may be due to differences in muscle insertion between the two sites, or structural functional adaptations of the tibia such as those involved in stress damping given the reduction in proximal trabecular bone^44,45^.

Paradoxically, *Celsr1* mutation mildly but significantly reduced eccentricity in the proximal tibia and around the tibia/fibula junction. Although the cellular or biomechanical bases for these differences are not known, they confirm that PCP signalling influences this parameter whereas, for example, the *Pax3*^*Sp2H*^ mutation does not. Differences in cross-sectional eccentricity have previously been reported in human infants with spina bifida^14^, although it is not known whether these patients had mutations in PCP pathway components. Unique sequence variants in both *VANGL2* and *CELSR1* have been previously identified in human spina bifida patients^46,47^, but to the authors’ knowledge skeletal structure has not been investigated in these patients. To our knowledge, all deleterious mutations in PCP genes associated with NTDs in humans reported to date have been identified in heterozygous patients, which is presumed to reflect multigenic aetiologies underlying these conditions^48^. Bone phenotypes identified in heterozygous mice in the present study may therefore be relevant to human NTD patients with heterozygous PCP mutations.

In the present study, mutation of *Celsr1* in the *Crsh* mice was found to be associated with more marked reduction in cortical bone mass than *Vangl2* mutation in *Lp* mice (i.e. 11.0% versus 3.3% reduction in Ct.Ar on average across all sites). A potential explanation for this is partial compensation for *Vangl2* by *Vangl1*^49^, although Vangl1 does not compensate for Vangl2 during neural tube closure^29^. Cortical thickness was particularly reduced around the tibia/fibula junction of *Celsr1*^*Crsh/*+^ mice, where tissue area was reduced (suggesting diminished periosteal expansion) whereas the medullary area was increased (suggesting greater endosteal expansion in these rapidly growing mice). The cell-specific roles of Vangl2 and Celsr1 in the osteoblast and osteoclast lineage are not currently known and merit investigation through targeted deletion. Nonetheless, the findings presented here confirm that PCP signalling enhances post-natal bone mass, particularly in the trabecular compartment.

In contrast, the heterozygous *Pax3*^*Sp2H*^ mutation had no significant effect on trabecular bone and minimal effects in the cortical compartment. Homozygous *Pax3* mutation results in NTDs and also diminishes embryonic muscle development, which is associated with skeletal dysmorphology^50^. The interpretation that skeletal phenotypes in homozygous *Pax3* mutants are secondary to reduced muscle formation^50^ is consistent with our findings that Pax3 is not expressed at detectable levels in osteoblast or osteoclast cultures at various stages of differentiation. Consequently, haploinsufficiency for Pax3 minimally altered tibial bone mass postnatally in male mice at the age investigated here despite these mice having detectable phenotypes in other organs; most notably the characteristic white ventral skin spot which gives the line its name^51^. Given our analyses of young male mice, we cannot exclude the possibility of deterioration (or indeed resolution) of skeletal phenotypes with age. Nonetheless, the young mice used in this study may be most relevant to paediatric cohorts. Fracture incidence in spina bifida patients, as in the general population, decreases in adulthood^10^.

As well as potentially contributing to a minority of spina bifida cases (at least in a Dutch population)^52^, *ZIC2* mutations are also directly associated with holoprosencephaly in humans^53^. We chose to study mutation of this gene because it causes fully penetrant spina bifida in mice^54^ and a polymorphism in it has previously been associated with reduced hip bone mineral density in a Chinese population^55^. Unexpectedly, we observed that tibial structure in *Zic2*^*Ku/*+^ mice was substantially different from their littermate controls in that their tibia/fibula junction was more proximally positioned. The genetic and/or environmental control of this anatomical landmark is not known, in part because it is not commonly analysed, reflecting an additional advantage of the global SSA approach we have used. In addition, *Zic2* mutation was associated with a significant reduction in cortical bone mass, site-specifically in the proximal region of the tibia. Intriguingly, this is the region of the tibia most responsive to axial mechanical loading^27^ and the associated transcription factor Zic1 translocates to the nucleus following mechanical stimulation of osteocyte-like cells *in vitro*^56^. Further work will be required to determine whether Zic transcription factors contribute to functional adaptation *in vivo*. An alternative mechanisms by which Zic2 may influence bone mass includes its known regulation of hedgehog signalling, which plays diverse roles in skeletal physiology and pathology^57^. Zic2 binds with the hedgehog pathway transcriptional regulator Gli and promotes its nuclear translocation^58^. Both Gli3 and Ptch1, another member of the hedgehog pathway, featured in the list of genetic commonalities between NTDs and bone. Zic2 has also recently been reported to be over-expressed in osteosarcoma cells^59^, in which it promotes viability and migration through a PI3K/AKT-dependant mechanism. Whether Zic2 promotes the same processes in non-neoplastic osteoblastic cells is not known.

Despite our findings that *Zic2*^*Ku/*+^*, Vangl2*^*Lp/*+^ and *Celsr1*^*Crsh/*+^ mice all have significantly lower bone mass than their WT littermates, none of these genes featured in an unbiased analysis of the known commonality between the genetic networks involved in NTDs and bone. This reflects the current lack of knowledge of these genes’ roles in bone, contrasting with the well-established roles of canonical Wnt signalling which substantially influences processes including osteoblast differentiation^60^ and bones’ functional adaptation to mechanical loading^61^. Specific Wnt pathway components are now important clinical targets for the treatment of osteoporosis^62^. Human syndromes associated with high or low bone mass caused by mutations in Wnt pathway components are not, to our knowledge, associated with NTDs. However, this is unsurprising given redundant expression of some Wnt pathway components during development (e.g. Lrp5 and Lrp6 redundantly control embryonic skeletogenesis^63^) and bone-specific expression of other proteins (e.g. of sclerostin primarily in osteocytes^64^). Wnt signalling was the most prominently enriched pathway in the NTD/bone overlapping genes list, which included canonical Wnt pathway components. Although PCP and canonical Wnt branches may be mutually antagonistic in other contexts^65^, both appear to promote bone mass in mice.

In summary, this study confirms extensive commonalities between molecular networks involved in successful neural tube closure in mice and those that determine bone structure. Our findings extend these commonalities to include two core components of the PCP pathway, Vangl2 and Celsr1, as well as Zic2. Detailed analysis of tibial structure revealed that mutation of either PCP component substantially diminished trabecular bone mass, whereas Zic2 mutation diminished cortical bone in a site-specific manner. Importantly, each of these effects was seen following heterozygous mutation of the gene in question. In as far as these findings can be extrapolated to humans, they suggest heterozygous mutations in these pathways may predispose to low bone mass even in spina bifida patients whose neurodevelopmental defect has multi-allelic bases^66^. Extension of these findings through human genetic and clinical studies may allow stratification of patients at high risk of fracture in spina bifida cohorts, as well as in other conditions associated with mutations in these genes, and determine whether they substantially contribute to fracture risk in the wider population.

## Methods

### Mouse models

Studies were performed under project license number 70/7469 under the UK Animals (Scientific Procedures) Act 1986 and the Medical Research Council’s Responsibility in the Use of Animals for Medical Research (1993) in accordance with the relevant guidelines and regulations. All analyses were performed on 21 day old male mice sacrificed at the time of weaning, representing a rapidly-growing “paediatric” stage. Mutant and WT littermates were always included from each litter, analysing n = 8 of each genotype. *Loop tail* mice carry a mutation in *Vangl2*^67^, *Crash (Crsh)* mice a mutation in *Celsr1*^29^, *Splotch* (*Sp*^2H^) mice a deletion in *Pax3*^68^, and *Kumba* (*Ku*) mice a mutation in *Zic2*^54^ as previously described. Although heterozygous mutation of the genes analysed occasionally result in NTDs, only unaffected mice were used in this study. As expected all *Vangl2*^*Lp/*+^, all *Celsr1*^*Crsh/*+^ and one of the *Zic2*^*Ku/*+^ mice had looped or kinked tails believed to reflect secondary neurulation defects, whereas *Pax3*^*Sp2H/*+^ mice have a ventral area of depigmentation associated with defective neural crest migration.

Mouse body weights and tibial lengths were not significantly different between any of the mutants and their WT littermates at the age investigated (Supplementary Tables 1–4).

### Bioinformatics analysis

Enriched gene ontology terms were identified using BiNGO analysis in Cytoscape^69^ as previously reported^70^. Interacting protein networks and KEGG pathway enrichments were identified and visualised using StringDB^71^. Network diagram links are based on experimental evidence and curated datasets, with line thickness indicating the strength of supporting data.

### µCT and Site Specificity Analysis

Tibiae were dissected immediately following euthanasia and dehydrated in 70% ethanol. Tibiae were scanned using a Skyscan 1176 micro-CT scanner (Bruker microCT, Kontich, Belgium). All scans were taken at 50 kV, 500 µA with 0.5 mm aluminium filter, and 0.4° rotation step. Individual 2D cross-sectional images were reconstructed using Bruker NRecon software version 1.6.10.2. Voxel resolution was 9 µm.

Trabecular analysis of the proximal tibia was performed by manually drawing around the trabecular region of interest as previously reported^20^, blinded to genotype. A 1 mm region starting 0.2 mm below the bottom of the growth plate was analysed and values for standard trabecular parameters are included in Supplementary Tables 1–4. Extensive cortical analysis was performed using SSA as previously described and validated^27^. SSA quantifies standard cross-sectional measures of bone mass and architecture at each one percent site of the bone’s length between 10% proximally and 90% distally. SSA data is shown as the mean ± SEM at each 1% site.

### Osteoblast and osteoclast cultures

Osteoblasts were isolated from the calvariae of 3–5 day old C57BL/6 mice by trypsin/collagenase digestion as previously described^72,73^. Cells were cultured for up to 21 days in alpha Minimum Essential Medium, (*α*MEM) supplemented with 2 mM β-glycerophosphate and 50 μg/ml ascorbic acid, with half medium changes every 3 days.

Osteoclasts were isolated from the long bones of 6–8 week-old C57BL/6 mice as described previously^74^. Cells were plated onto 5 mm diameter dentine discs (10^6^ cells) in 96-multiwells in αMEM supplemented with 10% FCS, 5% gentamicin, 100 nM PGE_2_, 200 ng/ml M-CSF and 3 ng/ml receptor activator of nuclear factor _Κ_B ligand (RANKL, R&D Systems Europe Ltd, Abingdon, UK). After 24 hours, discs containing adherent osteoclast precursors were transferred to 6-well trays (4 discs/well in 4 ml medium) for a further 6 days. Culture medium was acidified to pH~7.0 by the addition 10 meq/l H^+^ (as HCl) on day 7 to activate resorption^74^ and osteoclasts were cultured for a further 2 days.

### Total RNA extraction and DNase treatment

Osteoclasts were cultured on dentine discs for 7 (mature cells) or 9 days (mature, resorbing cells) before total RNA was extracted using TRIZOL^®^ reagent (Invitrogen, Paisley, UK) according to the manufacturer’s instructions. Osteoblasts were cultured for 7 (differentiating), 14 (mature) and 21 (mature, bone-forming) days before RNA collection. Extracted RNA was treated with RNase-free DNase I (35U/ml) for 30 min at 37 °C. The reaction was terminated by heat inactivation at 65 °C for 10 min. Total RNA was quantified spectrophotometrically by measuring absorbance at 260 nM. RNA was stored at −80 °C until amplification by qRT-PCR.

### Quantitative real time polymerase chain reaction (qRT-PCR)

Osteoclast and osteoblast RNA (50ng) was transcribed and amplified using the qPCRBIO SyGreen one-step qRT-PCR kit (PCR Biosystems, London, UK), which allows cDNA synthesis and PCR amplification to be carried out sequentially. qRT-PCR was performed according to manufacturer’s instructions with initial cDNA synthesis (45 °C for 10 min) and reverse transcriptase inactivation (95 °C for 2 min) followed by 40 cycles of denaturation (95 °C for 5 sec) and detection (60 °C for 30 sec). All reactions were carried out in triplicate using RNAs derived from 4–6 different cultures. Primer sequences (for/rev): **Vangl1**, acggagagtcccgcttctac/aattttccagaaccaccaagg: **Vangl2**, ccagccgcttctacaatgtc/tctccaggatccacactgc: **Celsr1**, ggcagtcatgaccttggacta/agctgattcccaatctgcac: **Scrib**, ccaccacggaagaagatgac/gttattggcctggtcaaacg: **Zic2**, tgcatgtccacacctcagat/gaggggaggactcatggac: **Pax3**, gcccacgtctattccacaa/gaatagtgctttggtgtacagtgc.

### Statistical analysis

Bioinformatics comparisons were based on the standard false discovery rate corrections applied by the software. Comparisons between two groups were by t-test and between three groups by one way ANOVA. SSA comparisons were by mixed model analysis in IBM SPSS Statistics 22 accounting from repeated measures at different sites from each mouse testing the main effects of genotype and site with a genotype by site interaction (indicating site-specificity of effects) as previously reported^27^. When the overall genotype effect was significant a Bonferroni post-hoc correction was applied to identify sites of significant difference. P < 0.05 was considered significant.

### Data availability

The datasets generated during and/or analysed during the current study are available from the corresponding author on reasonable request.

## Electronic supplementary material

Supplementary Figures
