# Supplementary material for: Spina bifida-predisposing heterozygous mutations in Planar Cell Polarity genes and Zic2 reduce bone mass in young mice
Source: Sci Rep. 2018 Feb 20;8:3325. doi: 10.1038/s41598-018-21718-x (PMC5820290; doi:10.1038/s41598-018-21718-x)
Supplement: Supplementary file 1 — Supplementary Figures [file 41598_2018_21718_MOESM1_ESM.pdf]

Spina bifida-predisposing heterozygous mutations in Planar Cell Polarity genes and Zic2 reduce bone mass in young mice

Isabel R Orriss[1], Stuart Lanham[2], Dawn Savery[3], Nicholas DE Greene[3], Philip Stanier[3], Richard Oreffo[2], Andrew J Copp[3], Gabriel L Galea[3,#]

1. Department of Comparative Biomedical Sciences, Royal Veterinary College, Camden, London, NW1 0TU, UK

2. Bone and Joint Research Group, Centre for Human Development, Stem Cells and Regeneration, Human Development and Health, Institute of Developmental Sciences, Faculty of Medicine, University of Southampton, Southampton, SO16 6YD, UK

3. Developmental Biology of Birth Defects, UCL GOS Institute of Child Health, 30 Guilford Street, London, WC1N 1EH, UK

# Corresponding author: Developmental Biology of Birth Defects, Wellcome Building W2.02, UCL GOS Institute of Child Health, 30 Guilford Street, London, WC1N 1EH, UK.

Email: g.galea@ucl.ac.uk

Keywords:

Bone, planar cell polarity, Vangl2, Celsr1, Zic2

Supplementary Figures and Legends:

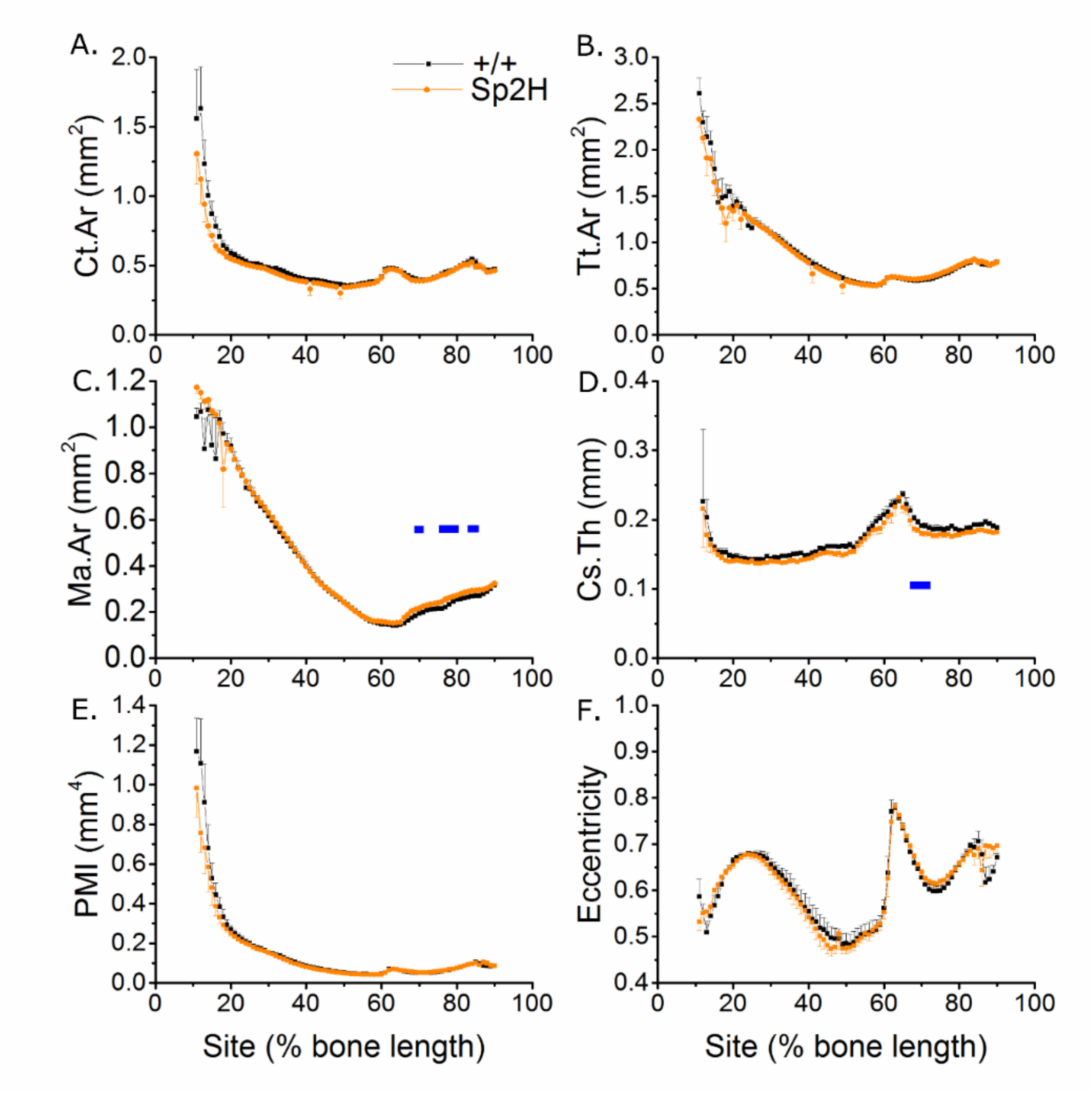

Supplementary Figure S1: Heterozygous *Splotch* mutation of Pax3 is associated with regionally smaller cortical bone mass in the tibia of young male mice. SSA µCT analysis of cortical bone structure at each 1% site along the bone's length in 3-week-old male Pax3$^{+/+}$ and Pax3$^{Sp2H/+}$ littermates (n = 8 each). A) Ct.Ar, B) Tt.Ar, C) Ma.Ar, D) Cs.Th, E) PMI and F) eccentricity were quantified. Blue lines indicate regions of significant differences between genotypes by mixed model analysis with Bonferroni post-hoc.

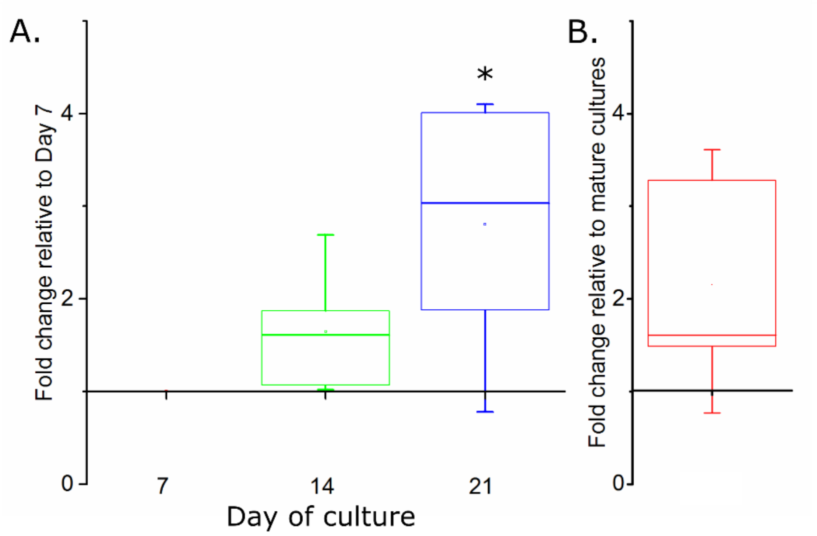

Supplementary Figure S2: Zic2 expression increases in mineralising osteoblasts. A,B) qRT-PCR quantification of Zic2 mRNA expression was quantified in (A) osteoblasts cultured for 7, 14 or 21 days and their expression compared to 7 day cultures (set at 1 for each set of cultures) or (B) mature resorbing osteoclasts (day 9 after 2 days of medium acidification) versus mature osteoclasts (day 7). N = 5-6, * $p < 0.05$

Supplementary Table S1: Trabecular bone parameters in Vangl2 *Lp* heteroyzgous mice and WT littermates

	Body weight (g)	Tibial length (mm)	BV/TV (%)	Tb.Th (mm)	Tb.Sp (mm)	Tb.N (/mm)	Tb.Pf (/mm)	SMI
**WT Mean**	12.825	13.872	12.787	0.064	0.494	2.000	13.186	1.889
**SEM**	0.496	0.129	0.932	0.002	0.025	0.114	1.512	0.062
**Lp/+ Mean**	12.575	13.726	8.306	0.058	0.499	1.417	20.731	2.243
**SEM**	0.297	0.103	0.982	0.002	0.025	0.153	1.505	0.060
**T-test p value**	0.672	0.406	**0.007**	**0.033**	0.895	**0.012**	**0.005**	**0.002**

Supplementary Table S2: Trabecular bone parameters in Celsr1 *Crsh* heteroyzgous mice and WT littermates

	Body weight (g)	Tibial length (mm)	BV/TV (%)	Tb.Th (mm)	Tb.Sp (mm)	Tb.N (/mm)	Tb.Pf (/mm)	SMI
**WT Mean**	11.538	13.350	18.216	0.071	0.430	2.572	10.775	1.861
**SEM**	0.235	0.095	0.882	0.002	0.017	0.087	0.991	0.036
**Crsh/+ Mean**	10.775	13.240	12.914	0.061	0.398	2.083	19.396	2.229
**SEM**	0.313	0.089	1.651	0.002	0.027	0.208	1.575	0.074
**T-test p value**	0.082	0.396	**0.047**	**0.019**	0.320	0.128	**0.002**	**0.002**

Supplementary Table S3: Trabecular bone parameters in Pax3 *Sp2H* heteroyzgous mice and WT littermates

	Body weight (g)	Tibial length (mm)	BV/TV (%)	Tb.Th (mm)	Tb.Sp (mm)	Tb.N (/mm)	Tb.Pf (/mm)	SMI
**WT Mean**	9.913	13.002	10.570	0.056	0.426	1.878	21.797	2.255
**SEM**	0.280	0.088	0.868	0.001	0.028	0.144	1.223	0.046
**Sp2H/+ Mean**	9.713	13.031	10.400	0.056	0.445	1.867	20.228	2.151
**SEM**	0.237	0.053	0.498	0.001	0.017	0.075	1.260	0.054
**T-test p value**	0.594	0.781	0.868	0.814	0.571	0.948	0.386	0.270

Supplementary Table S4: Trabecular bone parameters in Zic2 *Ku* heteroyzgous mice and WT littermates

	Body weight (g)	Tibial length (mm)	BV/TV (%)	Tb.Th (mm)	Tb.Sp (mm)	Tb.N (/mm)	Tb.Pf (/mm)	SMI
**WT Mean**	9.200	12.995	15.853	0.056	0.311	2.772	15.224	1.943
**SEM**	0.482	0.274	1.843	0.002	0.021	0.239	2.892	0.114
**Ku/+ Mean**	8.338	12.385	14.475	0.055	0.315	2.667	17.321	2.021
**SEM**	0.384	0.426	1.201	0.003	0.024	0.179	2.225	0.064
**T-test p value**	0.184	0.248	0.541	0.680	0.909	0.731	0.575	0.559

Supplementary Table S5: List of NTD-associated genes with "bone", "osteoblast" or "osteoblast" associated functional ontologies.

Msx2
Dlx5
Men1
Ptch1
Smad5
Gli3
Ski
Rab23
BMP7
BMP2
Nog
Nf1
Pitx2
Rarg
Rara
Csk
Fgfr1

Abi1
Abl1
Ptpn11
Lrp6
Twist1
Gja1
Cited2
Prkaca
Msx1
Tfap2a
Frem1
Tulp3
Hdac4
T
Ctnnbip1
Sfrp2
Wnt3a
Sfrp1
Cthrc1
Adar
Asf1a
Map3k7
Mapk8
Csnk1a1
Chuk
Trp53
Traf6
Rac1
Mapk9
